# Supplementary material for: Are carbon emissions trading and green financial instruments synergistic? -Comprehensive quantitative research based on content analysis
Source: PLoS One. 2024 Mar 7;19(3):e0298601. doi: 10.1371/journal.pone.0298601 (PMC10919601; doi:10.1371/journal.pone.0298601)
Supplement: S1 Data — (ZIP) [file pone.0298601.s001.zip › Data/地级市Z_F工作报告.zip!/╜¡╬≈/╥╦┤║╩╨/└┤╫╘╣1⁄2╓┌║┼í╛┬φ┐╦╩2╛▌═°í┐_2016.docx]

各位代表：

　　现在，我代表市人民政府向大会报告工作，请予审议，并请各位政协委员和其他列席人员提出意见。

　　一、2015年工作回顾

　　过去的一年，在省委、省政府和市委的正确领导下，我们围绕建设文明幸福宜春目标，紧扣“谋发展、比实干、争先进”要求，坚持稳中求进总基调，适应新常态，展现新作为，全市经济社会保持平稳健康发展，主要指标增幅高于全省平均水平，实现了“十二五”圆满收官。

　　——身膀更硬。总量增大：预计全市完成生产总值1620亿元，增长9.3%左右；财政总收入突破300亿元，达到308.8亿元，增长13.5%；规模以上工业增加值870.5亿元，增长9.4%，规模以上工业企业突破1000户，达1097户，净增144户；500万元以上固定资产投资1588.1亿元，增长17.2%；外贸出口148.1亿元，增长8.3%；实际利用外资6.5亿美元，增长11.9%；社会消费品零售总额530.7亿元，增长12.5%。质量提升：税收收入占财政总收入比重达84%，高出全省平均水平5.5个百分点；规模以上工业增加值、主营业务收入、利税总额均列全省前三。县域崛起：财政总收入超20亿元县市区7个，其中丰城突破60亿元，全国百强县再进5位、列78位；樟树突破50亿元，跻身全国最具投资潜力百强县；高安超35亿元，居全国县域经济发展潜力79位。

　　——形象更美。明月山旅游区晋升国家5A级，实现赣西零突破；宜春禅博园、宜丰九天度假区、樟树古海和[门][合]皂山获评国家4A级景区；《禅境宜春》获评最佳国际传播中国纪录片。成功创新中国成立家森林城市，摘取全省“平安杯”。举办锂电国际高峰论坛，亚洲锂都进一步叫响。全省生态文明先行示范区建设首个专项规划—《昌铜高速生态经济带总体规划》获批，昌铜四县列为首批示范县，占全省1/4。积极参加全国工商联执委会暨“名企助赣”系列活动，圆满承办第十三届赣台会，签约项目、金额均居全省前列。

　　——获得更多。收入稳步提高：城镇居民人均可支配收入25381元，增长9.3%；农民人均可支配收入11621元，增长10.4%。出行方便快捷：昌樟高速改扩建，昌栗、昌宁高速建成通车，宜慈公路一期基本建成，宜新公路即将通车。建成农村公路2311.5公里，新改建农村危桥58座，代表江西迎接“十二五”国省干线公路养护管理检查。住房条件改善：城市棚户区改造三年攻坚顺利推进，改造各类棚户区23705套、农村危房7108户，新开工公共租赁住房8239套。

　　一年来，我们主要做了以下工作：

　　（一）加速产业升级。着力壮大总量，优化结构，深入实施“三升级”战役。工业主导凸显。工业用电量110亿千瓦时，中国驰名商标41件，专利过千件园区3个，在建工业项目近500个，建材产业主营业务收入600亿元，锂电产业100亿元，均居全省第一。工业园区主营业务收入3300亿元，居全省第三；超亿元企业700余户，金源纺织、济民可信超60亿元；纳税千万元以上企业260户，四特酒达12亿元；宜春经开区增速居全省国家级开发区前三，锂电产业基地获全省优秀高新技术产业化基地；丰城、上高工业园区均突破500亿元。高新技术企业142户，省级以上创新平台54个，其中国家级3个，专利申请量3000件、授权1500件，均居全省前列。首座500千伏锦江变电站建成运营；获批筹建院士工作站6家，博士后科研工作站2家，实现企业建站零突破。推广新能源汽车1382辆，被评为全国新能源汽车推广应用示范市。樟树、丰城入选全国循环经济示范市，丰城获批第六批国家城市矿产示范基地，樟树、奉新分别荣获全国质量强市示范市、纺织产业集群创新发展示范区，上高、万载列为全国食品工业强县，高安建陶产业通过国家新型工业化示范基地评审。农业基础巩固。加快发展方式转变，农业总产值410亿元，粮食生产实现“十二连丰”，托市收购量全省第一。农村改革取得重大成果，土地确权颁证工作进度居全省前列；土地流转177万亩，占二轮承包总面积33%，铜鼓带溪发放全省首笔土地经营权抵押贷款。现代农业加快发展，新造高产油茶林11万亩，开发利用富硒农产品基地16.9万亩，肉牛出栏40.5万头，“财政惠农信贷通”发放贷款13.2亿元，均居全省第一；绿色食品原料基地123.6万亩，有机农产品81.4万亩，绿色有机认证产品496个，占全省40%；培育休闲农业经营主体431家；宜春国家农业科技园、丰城生态硒谷被评为国家农业科技示范园，袁州农业园区被列为国家农业综合开发试点，靖安、宜丰、铜鼓获批省级现代农业示范园，率先实现省级园区全覆盖，全省加快转变农业发展方式现场会在宜召开。146座小（２）型水库除险加固任务全面完成，基层国有粮食企业退城进郊工作扎实推进，新建仓容34.3万吨。上高获评全国粮食高产创建整县推进、农村“三资”管理示范县，靖安、奉新、万载分别被评为全国森林可持续经营试点县、平安农机示范县和首批有机产品认证示范区。服务业活力激发。以旅游业为龙头，推动现代服务业升级。接待游客3868.4万人次，旅游综合收入300.8亿元。明月山机场旅客吞吐量突破40万人次，创造了民航发展“宜春速度”。成功举办第九届月亮文化旅游节，同城自驾游活动创基尼斯纪录，明月山获评中国最佳温泉旅游目的地。新宜萍三市联合开通赣西旅游冠名高铁—宜春旅游号。奉新荣获全省旅游发展十佳县，靖安宝峰入选全国特色景观旅游名镇。在建服务业重大项目191个，投资额1273.2亿元，其中电商重大项目18个，交易额200亿元。“四导四创”电子商务孵化模式全省首创，宜春经开区物流园入选全国优秀园区；南氏电子商务产业园获批省级示范基地，370家企业入驻；阿里巴巴农村淘宝落户万载，开业当天创站点、订单和交易三项全国纪录。新增江西老字号企业12家，居全省第一。境外上市企业5家，“新三板”挂牌7家，均居全省前列。率先全省完成农信社改制，实现县县有自己的银行，新增贷款突破200亿元。

　　（二）统筹城乡发展。坚持以城带乡、城乡互动，城乡面貌焕然一新，城镇化率达44.5%，同比提高1.2个百分点。城建三年升级战圆满完成，累计投资1223亿元。首批19个示范（重点）镇村联动建设顺利收官，第二批13个镇（街道）全面启动，上高获评全国镇村联动干道改造提升工作先进县。率先全省编制完成行政村、县域村镇及中心村规划，丰城入选国家中小城市综合改革试点市，樟树临江列为国家建制镇示范试点。农村人居环境治理成效明显，生活垃圾处理县乡村组全覆盖，惠及438万农村居民。新农村建设扎实推进，旧村改造、空心村整治经验做法获省委、省政府肯定，高安梅江旧村改造、丰城空心村整治经验全省推广。注重集约节约用地，清理闲置土地2.2万亩，获批建设用地超5万亩，获评全省耕地保护成效显著设区市，上高被评为全省耕地保护责任目标考核先进县。在全省率先推进县级病死畜禽无害化处理中心建设，中心城区成为江西唯一入选全国首批生活垃圾分类示范市。奉新、上高成功创新中国成立家园林城，宜丰获批全国十佳生态文明城，靖安入选第二批全国生态文明示范工程试点。“十二五”减排目标全面完成。按照“零容忍”要求，查处环境问题企业546家，限期整改431家、责令停产59家、关停取缔56家，城区空气质量指数优良率全省领先。49座大中型水库全部实现人放天养，宜丰、靖安率先全省启动实施“河长制”，靖安被列为全国首批河湖管护体制机制创新试点。新增省级生态乡镇、村各7个。

　　（三）深化改革开放。按照“市场先导、管理放权、机制放活”要求，重点推进“1+N”改革措施。“三单一网”建设有序推进，在全省率先推出权责清单和便民服务事项责任清单，市行政服务中心入选全国百优单位。市政府机构改革圆满完成，事业单位分类、公务用车改革稳步推进。在全省率先设立预算稳定调节基金、编制全口径“四大预算”体系，建立跨年度预算平衡机制，财政透明度连续三年居全国前列。不动产统一登记改革全省靠前。丰城省直管县（市）体制改革试点、上高撤县设市工作稳步推进，高安“同类竞争、分类考核、打造效益政府”做法获中国政府创新优秀实践奖。主动“走出去、请进来”，招大引强取得新成果，引进5000万元以上项目295个、亿元以上174个，分别列全省第一、第二，万达广场、晶科电力等项目成功落户；对外直接投资8600万美元，超近9年总和。实施“宜商回归”工程，返乡创业项目117个，总投资180亿元。被省政府授予全省首个台湾青年创业基地，新增国际友好城市2个。宜春海关筹建稳步推进，高安铁路、万载公路口岸作业区建设基本完成，樟树水运口岸作业区建设进展顺利。

　　（四）完善保障体系。突出民生保障，公共服务不断完善。民生改善。新增城镇就业5.9万人，与新余、萍乡实现异地就医即时结算，被征地农民参加基本养老保险工作扎实推进。保险理赔12.7亿元，较好地防止了因灾因病致贫。在全省率先启动跨县区医疗救助网络同步结算、实施政府购买居家养老服务，荣获中国科学养老示范市。27.6万农村居民饮水安全问题得到解决。省级双拥模范城创建通过考评，“以村为单位，采取综合措施，实施精准扶贫”经验做法得到省委、省政府肯定。“十个一”志愿助残活动经验全国推广，宜丰、万载分别入选全国无障碍环境建设、农村社区建设实验全覆盖示范县，上高商城社区被列为全国综合减灾示范社区。市场物价基本稳定。事业进步。全面推进县域内义务教育学校校长教师交流轮岗，8个县市通过国家义务教育均衡发展评估认定，占全省1/3强。普通高考文、理科平均分列全省第一。新改扩建中小学18所，城区教育“大班额”问题有效缓解。宜春学院79级物理系学子陈仙辉、丰城籍科学家刘明获评中科院院士，奉新支月英入选“全国师德楷模”，“夺刀少年”柳艳兵、易政勇获全国道德模范提名奖。全民健身活动蓬勃开展，成为全省唯一的全国体育产业联系点。中心城古井保护工程顺利完成。市工商联获创新中国特别奖。广播电视人口综合覆盖率达99%。市档案馆、奉新文化馆晋升国家一级，上高被评为中国民间艺术之乡。乡镇“妇女之家”实现全覆盖，经验做法在全国妇联执委会上作典型发言。县级公立医院综合改革深入实施，市儿童医院投入使用，市人民医院（北院）建设扎实推进。低生育水平保持稳定。入选“中国好人榜”145人，居全国设区市前列。樟树、高安、宜丰获评全国基层中医药工作先进单位，奉新疾病预防控制中心被评为全国先进集体。社会稳定。注重解决群众合理诉求，信访工作取得新成效，袁州、宜丰、樟树连续四年荣获全省信访“三无”县市。便民代办服务延伸到组，市、县、乡、村四级服务体系更为完善。地质灾害预警和防治能力提升，连续7年实现人员零死亡。深化重点行业领域专项整治，健全食品药品安全体系，加强道路交通、消防安全监管，各类事故总量和死亡人数实现双下降，获评全省森林防火责任目标综合考评二等奖。构建立体化社会治安防控体系，严厉打击各类违法犯罪，公众安全感和满意度进一步提升。

　　（五）加强自身建设。深入开展“三严三实”专题教育，作风建设持续好转。认真落实“一岗双责”，党风廉政建设扎实推进。严格执行中央八项规定，“三公”经费压缩3.7%。法治政府建设成效明显，“六五”普法顺利完成，依法执政、依法行政体制机制不断完善，实现党政机关、企事业单位和人民团体法律顾问制度全覆盖。倡导“马上就办”理念，加大政府决议、抄告事项落实情况督查。开展企业、群众“评机关、评岗位”活动，发展环境进一步优化。自觉接受人大依法监督、政协民主监督和公众舆论监督，办结省、市人大代表建议108件、政协委员提案197件。强化行政监察，重点查处贪污挪用、截留挤占、骗取套取补贴资金等违纪行为。发挥审计监督“经济卫士”作用，配合做好市长经济责任审计，突出抓好政策落实和保障性安居工程等领域审计，完成项目119个，其中政府投资53个，核减工程造价4.2亿元。加强重大政策实施绩效评估，落实重大决策专家咨询、社会公示、听证、责任追究制度。主动公开政务信息8.7万条，组织在线访谈7期、新闻发布活动205场。国防后备力量建设、国家安全、外事侨务、民族宗教、邮政通信、烟草、盐业、气象水文、防震减灾、无线电管理、妇女儿童、统计和红十字会等工作均取得新成绩。

　　各位代表，本届政府自2011年成立以来，风雨无阻，日夜兼程，既感受到了奋斗的艰辛，又体会到了成功的喜悦，积累的认识和理念弥足珍贵，是全市人民的宝贵财富。

　　——知是行的主意，行是知的功夫；知是行之始，行是知之成。这是古代哲学家王阳明的经典论述，意思是只要有做一件事的念头，就是行的开始，而只有持之以恒的行动，才是实现理想的保证。我们坚持既知道更要做到，有念即行，知行合一。为加快县域经济发展，大力实施“双核两带”战略，健全差异化考核机制，丰樟高争当“排头兵”，致力发展循环经济；袁上万高举赶超大旗，突出做强绿色产业；昌铜高速沿线四县发挥优势，重点打造生态经济。为推进城乡一体化发展，强化“三高”理念，突出“四大一重”，中心城区框架拓展近一倍，镇村联动建设创造了城乡统筹的“宜春经验”。为促进旅游服务业升级，全面叫响“一年四季在宜春”，各地游客争相来宜“春看花、夏避暑、秋赏月、冬泡泉”。

　　——健康是前面的1，其余都是后面的0，项目也是如此，起着纲举目张、总揽全局的作用。抓经济工作，项目是根本、是主抓手，脱离了项目，只能是无源之水、无本之木。这犹如一个人，只有依托健康这个1，事业、家庭、财富等后面的0才能底气充足。我们完善了重大项目协调推进机制，累计召开现场办公会28次，解决各类问题500余个，促成了明月山机场通航，环城南路通车，320国道绕城改建开工，四方井水利枢纽工程有序推进，宜春人民30多年的梦想得以实现。武藏野、中国瑞林、奥其斯、紫宸科技等大批工业项目落地，为经济社会发展注入了源源不断的动力。

　　——莫道百姓可欺，既穿百姓衣，又食百姓饭，就谋百姓之事。我们谨记，权力是人民赋予的，为人民服务、为百姓办事是政府永恒的追求。先后实施了20个重点项目、双十工作、10+1工程和十大重点项目、50件民生实事，兴建了一批市民公园、运动游园、幸福书屋，加大了河流、农业面源等生态治理，解决了中心城区拆迁安置房产权登记、袁州干部同城不同酬等群众期盼已久的难题，用我们的辛勤指数换取了百姓的幸福指数。

　　各位代表，过去一年，在困难集中显现、风险相互交织的情势下，我们顶住了经济下行的压力，排除了前进道路上的障碍，赢得了加快发展的成果，“稳”的基础更加牢固，“进”的态势积极向好。成绩的取得，凝聚着全市人民的智慧汗水，是市委统揽全局、正确领导的结果，是市人大、市政协履职监督、共同努力的结果。在此，我谨代表市人民政府，向辛勤奋战在各条战线的广大干部群众，向给予政府工作大力支持的人大代表、政协委员、各民主党派、工商联、无党派、人民团体、驻宜部队、民兵预备役、武警官兵、公安民警和社会各界人士，以及所有关心支持宜春发展的同志们、朋友们，致以崇高的敬意和衷心的感谢！

　　在肯定成绩的同时，我们清醒地看到，宜春经济社会发展还存在不少矛盾和问题：产业集群化水平不高，缺乏旗舰式大企业、大项目支撑，尚未实现百亿企业、千亿产业零突破；城市管理有待加强，“地上”规范不够、“地下”标准不严；作风建设仍需改进，效率不高、推诿扯皮、缺乏担当等“不严不实”问题不同程度存在，等等。对于这些问题，我们将不回避、不遮掩，采取有效措施，切实加以解决，决不辜负全市人民的厚望!

　　二、2016年目标要求

　　各位代表，宜春历史悠久，人文荟萃，在这片神奇的土地上，风景如画，风调雨顺。解放以来，经过全市人民的不懈努力，依托资源禀赋，传统农业领跑全省，成为农村稳定、农业增收、农民致富的重要法宝，奠定了农业大市的地位；改革开放以来，乡镇企业异军突起，占据全市工业的“半壁江山”，揭开了宜春由农业主导向工业主导转化的序幕；新世纪以来，在中央和省委、省政府的关心支持下，宜春迎来“海陆空”时代，“铁公机”齐全，区位条件为之一变，为我们确立“三个中心”战略定位提供了前提；本届政府履职以来，树牢项目意识，坚持把项目作为经济工作的蓄水池和总装车间，用项目来落实省委、省政府改革开放的理念，用项目来推动各项事业的进步，为发展升级插上了腾飞之翅。只要我们接续奋斗、久久为功，宜春的明天将更加美好。

　　2016年是“十三五”规划的开局之年，也是五年决战同步全面小康的起始之年。做好今年的工作，既要正视宏观经济下行压力，又要看到经济持续向好的态势；既要正视发展不够的阶段性特征，又要看到转型升级是行稳致远的动力之源；既要正视财政实力、保障水平与民生需求的差距，又要守住民生底线，适应新常态，干出新作为。新常态是宜春的机遇。如果说常态下，沿海地区保持了高速发展，面对新常态，作为欠发达地区，“拖累点”相对较少，后发优势增多，可以抓住新一轮科技革命和产业变革机遇，自我加压，迎头赶上，实现“弯道超车”。新战略是宜春的引擎。“一带一路”、长江经济带、生态文明先行示范区建设、赣西经济转型加快发展等国家、省重大战略稳步推进，宜春将分享更多的政策红利。同时，供给侧改革的实施，以及中国制造2025、大众创业、万众创新等理念深入人心，新产业、新业态茁壮成长，“创时代”为宜春带来无限活力。新项目是宜春的底气。近几年来，通过努力争取，一批重点工程建成使用，一批产业项目投产见效，目前重大项目在库总数434个，其中投资10亿元以上186个，发展基础更加坚实，产业潜能进一步激发。只要我们保持定力，锐意进取，就能抢占制高点，把握主动权。

　　今年政府工作的总体要求是：全面贯彻落实党的十八大、十八届三中、四中、五中全会和习近平总书记“一个希望、三个着力”重要精神，遵循“四个全面”战略布局，紧扣省委“十六字”方针，把握稳中求进总基调，坚持创新、协调、绿色、开放、共享五大理念，以产业升级为主线，以项目建设为核心，以改革创新为动力，持续推进工业升级、农业升级、旅游服务业升级，促进县域经济发展、城镇化进程、城乡居民收入增速高于全省平均水平，进一步做大经济总量，做强县域板块，做活生态文章，做实民生保障，为确保与全省全国同步全面建成小康社会奠定坚实基础。

　　全市经济社会发展预期目标为：生产总值增长8.7%左右；财政总收入增长10%，其中一般公共预算收入增长10%；规模以上工业增加值增长9.2%；500万元以上固定资产投资增长15.7%；社会消费品零售总额增长11.1%；实际利用外资增长9.5%，外贸出口增长2.5%；城镇居民人均可支配收入增长9.2%，农村居民人均可支配收入增长10.2%；城镇登记失业率控制在4.5%以内，居民消费价格指数涨幅控制在3%以内。

　　三、2016年工作重点

　　今年是本届政府的收官之年，越是届尾的关键节点，我们更加不能守成、不得松懈、不可无为。要紧扣“十三五”目标，围绕全年任务，突出抓好以下六项工作：

　　（一）借助“互联网+”，实现产业升级新突破

　　以新型工业化为核心，协同推进现代农业和服务业融合发展，加快产业转型升级。

　　工业固本强基。深入实施兴工强市战略，突出集聚发展，培育八百骨干，做优丰樟高循环经济，做大袁上万绿色产业，做精昌铜高速沿线四县生态工业，力争七大支柱产业占比提高1～2个百分点。新增规模以上工业企业150户、主营业务收入超亿元800户、纳税千万元以上300户；园区主营业务收入3600亿元、十大省级重点产业集群1650亿元。运用物联网、云计算、大数据技术改造提升传统产业，培育壮大锂电新能源等新兴产业，着力打造世界最大的锂电池材料生产基地、亚洲最大的碳酸锂生产基地、中国重要的动力及储能锂电池生产基地、中部地区最大的新能源汽车生产基地。重点推进SK集团、正威国际、中节能等项目落地建设，支持济民可信、仁和、四特酒等骨干企业做大做强，引导金源、宝源、华春以及瑞麦、旺旺、必旺等整合重组，力争实现百亿企业零突破。

　　农业转型增效。坚持农业为农和产业化思维不动摇，致力转变发展方式，提高农业现代化水平。完善农村土地承包经营权确权登记颁证工作，推进成果应用改革试点，健全农业社会化服务体系。突出抓好油茶、肉牛、绿色有机、富硒和生态休闲五大产业，新造高产油茶林12万亩，肉牛出栏50万头，绿色食品原料基地140万亩，有机农产品100万亩，富硒农产品20万亩，新增省级以上休闲农业示范县1个、休闲农业示范点3～5个。提升现代农业示范园区建设水平，启动市本级休闲农业产业园建设。加强耕地保护，完成707.3万亩耕地保有量和617.7万亩基本农田目标。实施病险水闸除险加固、大中型灌区节水改造、小型农田水利和应急备用水源及配套设施建设，推进高标准绿色生态农田建设。

　　服务业优化升级。完善上下游产业配套，提升景区承载度，力争接待游客5200万人次、旅游综合收入430亿元，分别增长36.8%、43.3%。支持靖安三爪仑旅游区争创国家5A级，明月山创新中国成立家级旅游度假区、世界温泉健康名镇、中国温泉旅游名镇，加快温泉文化带、月之泉康疗中心、游客集散中心建设，推进袁州西海温泉、樟树四特酒文化城、丰城紫云山生态旅游景区、奉新九仙温泉、高安巴夫洛生态谷、铜鼓汤里温泉、万载古城·田下街区等在建项目，启动宜丰天宝古村及大潭山次中心旅游项目建设，筹办全省旅游产业集群现场推进会。加快发展电子商务，做大连锁经营、网络运营、物流快递等新型服务产业，争取社会消费品零售总额560亿元、电商交易额增长50%以上，创建省级电商示范基地和示范企业3个。加大金融机构引进力度，鼓励更多企业上市。

　　（二）加快“市民化”，构建城乡一体新格局

　　坚持把城镇当城市建、农村当社区管，促进城乡同发展、共繁荣。

　　打造区域中心。采取“多规合一”的形式，实施中心城总规修编。完成320国道绕城改建工程，启动环城东路改线暨渥江湿地公园建设，促进财富广场、红林世界城、赣西汽车服务广场、跨沪昆高速立交互通、万达广场等项目建设。完善老城区路网体系，实施人行天桥、地下通道及部分主干道改造，强化地下人防工程规划建设与应用，增购新能源公交车100辆，加快公共自行车租赁系统建设，缓解城区拥堵问题。推进海绵城市建设，拓展“互联网+”便民服务功能，搭建视频图像联网共享平台。以国家卫生城复评迎检为契机，以联创联建、全民卫生日为抓手，创新体制机制，推行数字城管，提升城市管理水平。精选1～2条街区进行灯光、色彩设计，有效改善市容市貌。健全分析预警机制，加大去库存力度，促进房地产市场平稳发展。

　　推进镇村建设。巩固首批镇村联动建设成果，实施第二批“30+”项目，抓好全国重点镇、全省百强中心镇建设，支持县市申报新型城镇化试点。建设中心村63个，推进“七改三网”和“10+4”等配套工程。启动集镇污水处理三年行动试点，健全垃圾收运处理体系，力争昌铜高速沿线四县率先通过省考核验收。

　　完善基础设施。做好城乡统筹发展前期规划，促成明月山机场扩建、四方井水利枢纽工程开工、靖安洪屏抽水蓄能电站并网发电，推进丰电三期、大唐集团上高电厂等建设，实施城镇电网改建、农村路网升级和危桥改造，加快到组公路建设，确保铜万、东昌高速公路通车。配合做好昌吉赣客专和蒙华铁路煤运通道征地拆迁，争取宜春火车站升级为一等站，启动中心城区生活垃圾综合处理利用工程建设。

　　（三）打好攻坚战，迈出改革开放新步伐

　　全面落实“规定动作”，大胆探索“自选动作”，不断释放改革红利。

　　深化改革。加快户籍制度、农业农村、城乡公交一体化、医疗卫生体制及教育领域综合改革，推进公共服务常住人口全覆盖。确保不动产统一登记“新开旧停”。深化财税和公共资源交易体制改革，全面推动中期财政规划管理，有序实施“营改增”改革。抓好事业单位分类改革，完善“三单一网”管理，不能止步于纸上，而要成为群众便捷办事的“心愿清单”。

　　招大引强。围绕锂电新能源、生物医药、绿色食品等优势产业，开展特大型项目攻坚活动，引进一批具有支撑引领作用的龙头企业。促进央企入宜、名企入宜、外资入宜，争取引进国内外500强项目10个、50亿元以上5个、10亿元以上50个。加强宜台文化交流，促成更多台资企业落户。

　　扩大开放。做好宜春海关筹建工作，力争下半年开关，建成高安、万载口岸作业区，推进樟树水运口岸、丰城海铁联运陆地港建设，开通宜春至宁波的接替式五定班列，启动宜春航空物流园建设。加强与九江、莆田等地口岸物流的对接合作，打通物流新通道。主动走出去，做好金属家具、建筑陶瓷、农产品等传统优势产业的出口培育，支持企业境外设立研发中心，扩张营销网络。以非洲、澳洲、“一带一路”沿线国家为主要市场，开展对外投资和工程承包业务。

　　（四）培育众“创客”，激发创新创业新动力

　　致力疏通创新创业中的“堵点”，破解创新创业中的“难点”，进一步激发社会活力、释放巨大动力。

　　降低门槛。落实创新创业33条，促进“先照后证”等注册资本登记制度改革，实施“三证合一”、“一照一码”，为创新创业提供最大便利。启动独立科研院所科技人员创新创业试点，鼓励高校、科研院所等事业单位专业技术人员在岗或离岗创新创业。推进“宜商回归”工程，每个县（市、区）创建回乡创业园1个以上。推广大学生创业引领计划，至少扶持500名大学生成功创业。

　　创新驱动。实施产业转型升级关键技术集中攻坚，加快高安建筑陶瓷、樟树金属家具国家品牌示范区建设，力争新开发省级重点新产品180个，新增高新技术企业45户、专利申请量700件以上，中国驰名商标10个。鼓励宜春学院、宜春职业技术学院建立创业学院、创新中心、创客空间，争创大学生创业孵化省级基地。加强对外交流，巩固与武汉大学、同济大学、中南大学等高校及科研院所合作，促成产学研项目200个以上。

　　优化环境。深植创新创业文化，推行行政审批零障碍、委托代办零收费、配套接入零负担、生产经营零干扰、各类服务零距离“五零”承诺，营造鼓励创业、宽容失败的良好环境。开展降低企业成本行动，促进工商业用电用气同价，精简涉企收费项目，减轻失业保险缴费负担。支持阿里巴巴“农村淘宝”项目在宜春扩大试点，培育发展五洲医药、捷一商务、金农米业等电子商务企业，做强南氏电子商务产业园和宜春众购网络平台，力争销售额突破20亿元。探索绿色信贷投入机制，推广“两权”抵押贷款，继续推进“财园信贷通”、“财政惠农信贷通”融资。

　　（五）打造高“颜值”，彰显绿色生态新优势

　　珍惜绿色财富，放大生态优势，做到保护与治理同步，发展与绿色同行，让“宜春蓝”永驻。

　　壮大经济。推进昌铜高速沿线四县省生态文明先行示范县建设，实施“金桥、金牌、金山”工程，努力把昌铜高速生态经济带打造成宜春的名片、江西的样板、全国的亮点。编制丰樟高循环经济发展和袁上万绿色发展规划，支持丰樟高做大循环经济，袁上万做强绿色产业。有条件的县（市、区）争取国家木材战略储备、特殊林木培育等项目。创新森林经营理念，探索发展林下经济，将森林资源优势转化为经济优势。

　　加强创建。总结锦江流域整治经验，推进袁河、肖江、潦河等重点流域及饮用水源地、水库水环境综合治理，杜绝劣五类水质。加强矿山地质环境保护，完成人工造林23.9万亩、封山育林10.3万亩、森林抚育42.1万亩。支持县（市、区）创建省级森林城，丰城、奉新分别创国家文明城、生态文明县，靖安创省水生态文明示范县和城乡生活垃圾分类一体化试点，宜丰、铜鼓创省园林城，袁州、上高创省生态文明先行示范县。加快生态文明村风景林建设。加强外来有害生物防范。

　　坚守底线。全面启动重点生态功能区、生态环境敏感区和脆弱区生态保护红线划定工作，探索管制措施。持续开展“零点行动”，抓好重点行业、重点企业环境管控。加强PM2.5监测，加大渣土扬尘、机动车尾气等治理，促进工业园区污水管网建设，做好“黄标车”淘汰工作。健全江河湖库管理体系，打造治理样板，实现“河长制”管理全覆盖。注重农业面源和养殖污染整治，全面建成县级病死畜禽无害化处理中心。淘汰落后产能企业15家以上，单位工业增加值能耗下降3%以上。

　　（六）增强获得感，推动民生福祉新改善

　　任何事情，从现在做都不晚。我们既要追求看得着、摸得到的“硬政绩”，也要提供让群众得实惠、谋长远的“软服务”。

　　完善保障。拓宽就业渠道，新增城镇就业4.7万人，转移农村劳动力6.7万人。推进精准扶贫，扩大小额扶贫信贷规模，力争脱贫3.5万人以上，贫困村农民人均收入达到8500元。推广居家养老服务“袁州模式”，覆盖城市社区50%、农村社区25%。打好城市棚户区改造三年攻坚，完成各类棚户区改造13110套。探索租购代建模式，解决1700户群众住房问题。完善城乡社会保障体系，扩大参保缴费覆盖面。实施城乡居民大病保险，健全重特大疾病保障机制。推行“助保贷款”政策，落实机关事业单位养老保险制度改革和被征地农民养老保险待遇，建立困难残疾人生活补贴等制度。推进农村安全饮水工程，保障312.8万人口饮水安全。抓好“菜篮子”工程，优化便民蔬菜直销店布局，加强农贸市场环境整治。理顺食品药品监管体制，开展“双打”、“质监利剑”等专项行动，维护市场正常秩序。

　　繁荣事业。实施“三通两平台”建设，推进现代职业教育，提升中小学办学品质，提高高中阶段教育普及率，促进各类教育协调发展。抓好中小学校新改扩建，有效缓解城区教育“大班额”问题。弘扬“好人文化”，争创省第五届文明城市。扶持发展文化创意产业，争创省级示范基地5～6个。支持市属媒体应用新技术，发展新媒体，加快融合发展。加强基层群众文化活动场所建设，提高公共文化服务能力，力争社区有室外活动场地，街道有室内活动中心，主要群众文化团体有活动场所。抓好世界文化遗产申报，加强吴城、筑卫城、樊城堆遗址保护，启动中心城历史文化街区保护工程和美术馆建设；促成811台迁建，加快禅宗祖庭恢复重建，完成26集禅宗文化动画片制作。健全妇幼医疗保健服务体系，落实全面二孩政策；探索建立分级诊疗制度，提高基本医疗保障水平。完善社区和乡村运动场所规划，兴建市民体育运动中心，筹备全国农耕健身大赛和市第五届全民健身运动会。建成市儿童活动中心，推进数字档案馆建设。

　　维护稳定。落实安全生产“党政同责、一岗双责、失职追责”责任制和企业主体责任，加大隐患治理，严防重特大安全事故发生。开展领导开门接访和带案下访活动，促进信访积案化解。强化金融监管，加强诚信建设，加大非法集资打击力度。完善公共安全和应急管理机制，提高预警处置能力。抓好防灾减灾救灾各项工作，促进国防动员、民兵预备役、人民防空建设。完善立体化社会治安防控体系，推进“天网”、“地网”和“技防入户”等智慧平安建设，加强反恐和禁毒工作，严厉打击各类违法犯罪，不断提升群众安全感和满意度。

　　各位代表，我们要积极回应群众的普遍关切，突出抓好十类民生项目100件实事，让全市人民共享发展成果。进一步加强自身建设，践行“三严三实”，努力打造人民满意政府。依法依规，提升公信力。深入推进法治宜春建设，树立法治思维，做到依法履职、规范用权。普及法律顾问制度，聘请法律“先生”，把好法治“闸门”，确保作决策、抓工作于法有据。完善法律援助制度，落实政府法律援助责任。自觉接受人大依法监督、政协民主监督，认真办理人大代表建议和政协委员提案，提升政府决策水平。务实落实，提升执行力。所谓行政，行动才有政绩，落实才会政成，定下的事雷厉风行，看准的事一抓到底。创造性执行，主动性服务，遇到问题多想办法、少找理由，多下功夫、少讲托辞，做到工作快上手、措施快跟进、服务快节奏，扎实有效推进各项工作。勤政廉政，提升向心力。牢固树立纪律和规矩意识，落实党风廉政建设“一岗双责”，筑牢拒腐防变大堤。严格执行中央八项规定，严控“三公”经费，把有限的资源和财力，用到发展最急需的地方，用在民生最急迫的事情。加大监察、审计、财政监督力度，强化对重大工程、重点行业和重要环节的监管，严肃查处各类违纪违法案件，铲除“权力寻租”空间。

　　各位代表，既然选择了方向，就要不畏风雨；既然接受了重托，就要不辱使命。让我们在市委的坚强领导下，紧紧依靠全市人民，强化担当，主动作为，为实现“十三五‘提前翻番、力争百强、同步小康’”的宏伟目标而努力奋斗！
